# Stability Properties of Underdominance in Finite Subdivided Populations

**DOI:** 10.1371/journal.pcbi.1002260

**Published:** 2011-11-03

**Authors:** Philipp M. Altrock, Arne Traulsen, Floyd A. Reed

**Affiliations:** 1 Evolutionary Theory Group, Max-Planck-Institute for Evolutionary Biology, Plön, Germany; 2 Population Genetics Group, Department of Evolutionary Genetics, Max-Planck-Institute for Evolutionary Biology, Plön, Germany; 3 Department of Biology, University of Hawai'i at Mānoa, Honolulu, Hawaii, United States of America; University of Gothenburg, Sweden

## Abstract

In isolated populations underdominance leads to bistable evolutionary dynamics: below a certain mutant allele frequency the wildtype succeeds. Above this point, the potentially underdominant mutant allele fixes. In subdivided populations with gene flow there can be stable states with coexistence of wildtypes and mutants: polymorphism can be maintained because of a migration-selection equilibrium, i.e., selection against rare recent immigrant alleles that tend to be heterozygous. We focus on the stochastic evolutionary dynamics of systems where demographic fluctuations in the coupled populations are the main source of internal noise. We discuss the influence of fitness, migration rate, and the relative sizes of two interacting populations on the mean extinction times of a group of potentially underdominant mutant alleles. We classify realistic initial conditions according to their impact on the stochastic extinction process. Even in small populations, where demographic fluctuations are large, stability properties predicted from deterministic dynamics show remarkable robustness. Fixation of the mutant allele becomes unlikely but the time to its extinction can be long.

## Author Summary

Underdominance is a component of natural evolution: homozygotes – of either wildtypes or mutants – are advantageous. This can play a role in speciation and as a method to establish artificial genetic constructs in wild populations. The polymorphic state of wildtype and mutant alleles is unstable. However, in subdivided populations limited gene flow can counterbalance this effect. The maintenance of polymorphism sensitively depends on the amount of gene flow. In populations of finite size, the polymorphism is ultimately lost due to stochastic fluctuations, but there are long intermediate periods of polymorphism persistence. We analyze a simple population genetic model to characterize and explore the polymorphic phases depending on population size and genotypic fitness values. Even for large fluctuations (small population size), long periods of neither extinction nor fixation are possible. Since underdominance has been proposed as a genetic strategy in the pest management of disease vectors, it is important to understand the basic features of this system precisely, especially with a focus on gene flow between ecological patches. We assess different release strategies of potentially underdominant mutants, where one seeks to minimize the probability of fixation of the introduced allele but maximize the time to its extinction.

## Introduction

A population can evolve due to differences in relative reproductive success over a life cycle. Fitness, in an evolutionary genetic sense, is defined as the relative expected number of descendants in the next generation based on an individual's genotype. In diploid organisms, two alleles can result in three genotype combinations, two homozygous genotypes with two copies of the same allele, and one heterozygote type with one copy of each allelic type. Heterozygote disadvantage in reproductive success is termed underdominance: Heterozygous individuals have a lower relative fitness than both homozygotes. The fundamental properties of underdominance in large populations with deterministic dynamics are well known [1], [2], [3]. Underdominance acts as an evolutionarily bi-stable switch. A mutant allele that is in underdominance with the wildtype is expected to be lost if its initial frequency is below a certain threshold. However, if the initial frequency is above this threshold frequency, it can also proceed to fixation. The threshold frequency is determined by the fitness values of the genotypes involved [4], [5]. The evolutionary dynamics induced by underdominance are similar to those in a coordination game, such as the stag hunt [6], [7], [8], [9], [10].

Under natural conditions underdominance can be caused by chromosomal rearrangements [11]. These rearrangements can accumulate between closely related species [12], [13], despite an exceedingly small predicted probability of becoming established [14], [15]. Individuals that are heterozygotes for a reciprocal translocation suffer from reduced fertility compared to homozygotes. This is due to a disrupted number of gene copies in the affected chromosomal region (i.e. segmental aneuploidy) [16].

We focus on the dynamics of a single locus with underdominant alleles of large fitness effects, such as those expected with natural reciprocal translocations. There has also been research into multiple loci of weaker individual effects, which can have interesting self-organizing properties [17], [18]. Alternatively, ‘engineered underdominance’ approaches based on reciprocal suppression of toxic constructs have also been proposed, which have much lower thresholds for a population transformation than typically expected [19], [20]. Finally, frequency dependent interactions can have underdominant-like properties, such as maternal-effect chiral dynamics in snails [21], and the Rh factor in humans [22]. However, details of these additional cases are beyond the scope of the work described here. Our results apply to classical single locus underdominance with large fitness effects.

As an artificial genetic construct, underdominance has been proposed as a method to stably establish linked alleles with desirable properties in the wild; for example, rendering insect populations resistant to diseases that otherwise can be transferred to humans (or other species), such as malaria or Dengue fever [23]. The bi-stable nature of the evolutionary dynamics suggests that a sufficient release of transformed individuals will ultimately result in complete fixation of the transformed allele in the population. Additionally, the system is reversible: A release of a sufficient number of wildtypes can bring the population back to its original state.

Underdominant polymorphism is eventually lost or completely fixed in single populations. However, it is known that it can become stable at mixed frequencies due to a migration-selection equilibrium in large subdivided populations that exchange a fraction of migrants [24], [25], [26]. An underdominant polymorphism can be maintained if the migration rate is below a bifurcation point, which depends on the genotypic fitness values [26]. Higher migration rates result in sufficient mixing, such that the two population system effectively reduces to a single population and the polymorphic state is lost.

Initial testing of genetic pest management systems is likely to take place on more isolated physical or ecological islands [27], [28], [29]. Furthermore, there are potential conservation applications of this type of technology in many island species [30]. However, smaller insect population sizes on islands may not be well approximated by deterministic dynamics based on an infinite population assumption. Here, we extend the understanding of the evolutionary dynamics of underdominance in two demes to include stochastic effects in finite populations.

Generations are overlapping in species that do not strictly follow discrete time reproductive patterns. Hence, we concentrate on Moran models describing the stochastic invasion and fixation of transformed or mutant alleles in a system of coupled populations. A Moran process considers a single reproductive event in one time step such that after 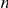 time steps in a population of fixed size 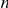, each individual has reproduced once on average. If the timescales are small enough that further mutations can be excluded, loss or fixation of a given allele are the only possible outcomes. As a simplification to our stochastic model, we assume that the two populations exchange migrants at the same rate. How likely are extinction or fixation of a certain number (release) of genetically transformed mutant alleles? A release strategy can be defined by number of released individuals and the release fractions in each sub-population. How long can we expect a successfully transformed local population to maintain the modified allele? How robust is the notion of stability from the deterministic system in the presence of fluctuations? We are also interested in population size asymmetry, where a simplified island-continent model can be appropriate.

The manuscript is organized in the following way. The next part of this section briefly repeats aspects of evolutionary dynamics in two infinitely large populations coupled by migration. Then, we introduce our model based on a Moran process for two populations with migration. The section ends with the introduction of a one-dimensional island continent model, which directly follows as a solvable limit case. In the Models section we first give the precise formulation of the discrete stochastic dynamics in two dimensions and argue how to access its properties by simulations. Secondly, we derive the island-continent model, which allows a prediction for the mean extinction times of the mutant allele in a small island population. In the last section, all results are discussed, followed by a concluding summary.

### Replicator dynamics

With 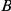 we denote the wildtype allele, whereas 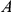 represents a transformed (or mutant) allele. Given a single locus two allele model of diploid organisms, there are three genotypes possible: 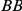, 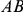, and 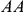. We set the average allelic fitness of wildtypes (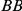) to 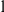, the fitness of heterozygote genotype (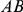) to 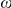, and the fitness of homozygous mutants (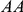) to 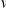. The fitness ordering 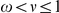 leads to underdominance. Under random mating, we can describe the population by the frequencies of the alleles (i.e., random union of gametes predicts the relative abundance of initial zygotic genotypes in the population before applying selection). For allele 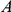 with relative abundance 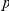 in a single population, the average fitness is then given by 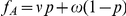. Likewise, for the wildtype allele 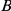 we have 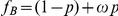. In general, for overlapping generations, a replicator equation describes the change in allele frequency in an infinitely large (well mixed) population in continuous time,
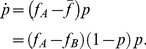
(1)Here, 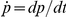 denotes the temporal derivative and 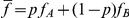 is the average fitness of the population. The roots of Eq. 1 give the fixed points 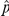. In the case of underdominance, 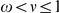, we have the stable fixed points 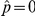 and 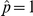 as well as the unstable fixed point 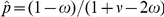.

For two local populations that exchange migrants we introduce the rate of migration 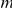 as a macroscopic parameter. In a small time interval 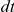, the fraction of immigrants is 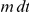. Hence, 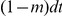 is the fraction of non-migrant individuals. Let 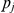 be the frequency of allele 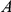 in population 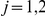. With the flow of alleles from the other population due to migration, the frequencies that contribute to the change in 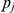 over time are 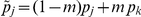, where 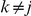, in both populations. The total average fitness in either population is 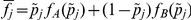. Hence, the replicator equation for the coupled system (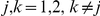) reads
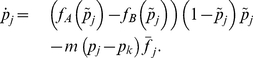
(2)These dynamic equations follow from Eq. 1, 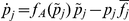 [26]. The number of fixed points and their stability properties depend on the rate of migration. The points 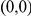 and 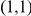 are always stable. Migration has no effect on the diagonal 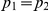. Exchanging alleles between populations at equal frequencies results in no change in either of them. The point 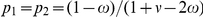 on the diagonal is an unstable fixed point, reflecting the inner unstable point of the single population case. For symmetric underdominance, 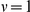, and 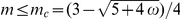, there are two stable states in the interior of the joined allele frequency space, i.e. where 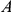 is neither fixed, nor lost. These stable fixed points of the dynamics are located on the symmetry axis, 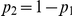. For general fitness values 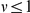, this symmetry is broken, but such internal stable equilibria can still exist below a critical migration rate 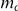 [26].

### Moran process

We focus on a Moran model with fixed population sizes. Our main assumption is that mate choice is random. In this case individuals in the population can be thought of as passing through the Hardy-Weinberg expectations at some point in life-cycle before selection. Hence, we can consider the system as if individual alleles (i.e. gametes) reproduce and die. Reproduction is proportional to fitness and death is random. Such discrete stochastic birth-death processes are typically used to describe the (transient) microscopic evolutionary dynamics in single uncoupled populations of finite size [31], [32], [33], [34], [35], [36]. From the microscopic dynamics, one is interested in macroscopic quantities such as the probability of extinction, and the associated extinction times.

Our two populations are of size 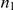 and 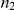. The number of individual copies of the mutant allele 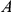 (type 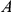) in each population are 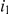 and 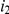, jointly defining the state. Thus, type 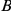 has frequencies 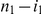, 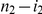, respectively. As we are concerned with diploid organisms the total number of alleles in each population is 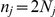, where 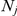 is the number of organisms in population . Time is scaled in units of half the time between organismal reproduction events, i.e. the time between individual allele reproduction events. For convenience we introduce the fractions 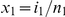 and 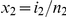. The average allelic fitness functions are
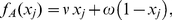
(3)

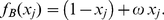
(4)For a consistent stochastic model several events have to be considered independently in one time step of the Moran process.

**Figure 1:**
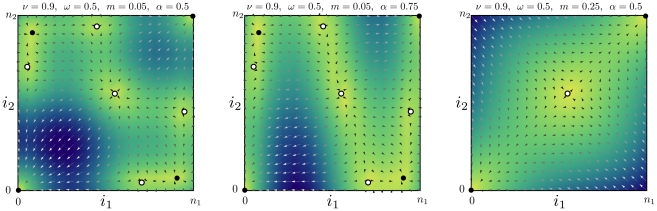
Direction of selection in the two population system with migration. We show a phase portrait of the gradient of selection with 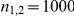. The arrows (length rescaled) indicate the most likely direction of selection given by Eqs. 6–9. The shading indicates the average speed of selection: The darker she shading, the faster the system is expected to leave the given state. Stable fixed points of the replicator dynamics are given by filled disks. Unstable fixed points and saddles are denoted by empty disks. **Left panel**: The migration rate is below the critical value 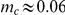, such that the replicator dynamics has internal stable fixed points. The number of alleles changes equally fast in both populations . **Central panel**: For the same migration rate, but with one population changing three times as fast compared to the other (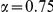), the selection pattern changes. However, the fixed points of the replicator dynamics Eq. 2 remain the same. **Right panel**: The stability of the fixed points of the replicator dynamics changes critically with the migration rate 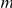. For sufficiently high migration rate, 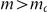, the system proceeds fast to fixation or loss of the mutant allele.

First, with probability 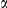 a reproductive event occurs in population 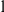. With probability 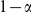 a reproductive event occurs in population 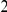. We exclude simultaneous reproductive events in both populations and treat the two population system as one Markov chain with the two absorbing states 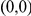, and 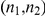. One population may change more rapidly than the other (i.e. more events occur in the larger population per unit time). If 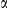 is the relative reproductive rate under neutrality (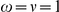), we have 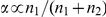, and thus 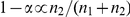. Hence, for the study of two populations of comparable size, it is convenient to set 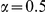. The choice of 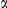 does not change the migration-selection equilibria predicted by the replicator system Eq. 2, compare Fig. 1. Only the rates of change between fixed points are increased in larger populations.

Secondly, in population 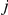, the number of alleles of either type increases with a probability proportional to the average fitness of the allele. In such an event, however, we have to consider that with probability 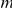, the parent individual allele is from the other population (i.e., an immigrant). Hence, type 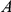 produces an identical offspring with probability proportional to 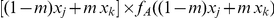. A similar probability holds for type 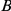 offspring, 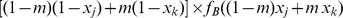.

Thirdly, in each population, the total number of alleles is held constant. This implies that for each birth event, there is an independent death event: a randomly chosen individual allele is removed from the population. A type 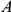 allele is removed with probability 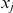, a type 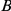 allele is removed with probability 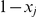.

**Figure 2:**
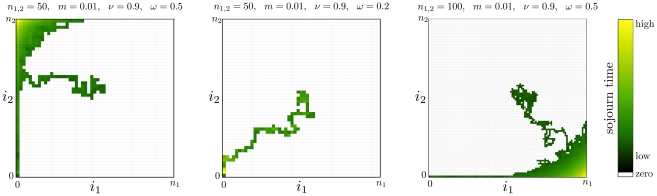
Stochastic evolution of the mutant allele in two coupled populations. Typical trajectories for the loss of the mutant allele (extinction process) in a system of two populations of the same size, 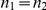. We show different realizations of the two dimensional Markov chain. The initial condition is the unstable equilibrium near the center 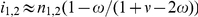, the final state is 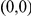 in all three cases. The shading indicates the sojourn time (total time spent in a particular state, including waiting times). The brighter the shading, the more often the respective state has been visited, white states were not visited. **Left panel**: Typically, the process spends long times near the 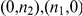 corners, where the waiting times are highest. **Center panel**: The process proceeds fast to extinction of the mutant allele, but slows down near 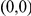. **Right panel**: The process spends most of the time in the 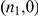 corner. Once it proceeds to extinction, it moves fast along a boundary of the allele frequency space, i.e. the mutant allele does not invade the other population again.

Overall, given the state 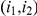, there are five events possible. Four of them involve a change in allele frequency 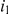, or 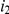. Hence, we have to define four transition probabilities in each state, 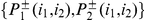, such that migration and selection only contribute to birth and not to random death. In general, fixation or loss in both populations are the only absorbing states, i.e. 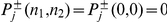. Due to migration, there is a non-vanishing flow perpendicular to the boundaries in state space. When the mutant allele 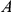 is lost or fixed in only one population, immigrants can drive the system back into the interior, where 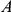 is present in both populations, compare Fig. 1 and Fig. 2.

### Limiting cases

Let us first consider an island and continent situation. On the large continent, migrants from the small island introduce 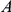 at a very low frequency. The wildtype allele is fixed and allele 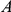 cannot invade by migration. However, there is a non-vanishing fitness contribution due to migration to the island, which receives wildtype immigrants from, and loses migrants of any type to the continent. This can be described by the limit case of the two population system where one population becomes infinite and the other remains finite. Given the fitness functions Eqs. 3 and 4, an equivalent limit case is 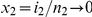. Applying this limit to the transition rates , the single stochastic variable becomes 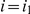, and time can be rescaled such that 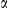 drops out. This yields a one-dimensional birth-death process on 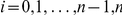. The one-dimensional transition probabilities are 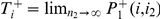 and 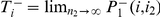. Here, 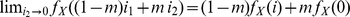, (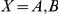), where 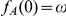, and 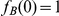. The continent can only contribute to the birth of wildtype homozygotes. Thus, 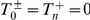, and 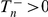.

In the Models section we show how the moments 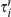 of the extinction times associated with the extinction process on the island starting with 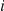 mutant alleles can be determined. The probability that allele 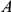 ultimately vanishes in the island population is 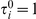. For 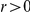, the 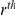 moment follows recursively from
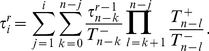
(5)Of most interest is typically the mean life time, or mean extinction time, of the allele 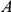, 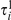.

Another case that leads to one-dimensional evolutionary dynamics is the limit of high migration rate, such that the two populations become genetically indistinguishable. This yields slightly different dynamics in a population of 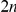 individuals, namely a one-dimensional Markov chain with two absorbing boundaries. For such processes the extinction/fixation times are formally well understood [31], [37]. The expression for the mean extinction time of a mutant allele at frequency 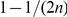 is similar to Eq. 5 with 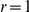, 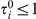 [33].

## Models

### Moran process for two coupled populations

With migration the number of 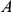 alleles in each population is 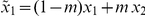, and 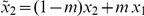. Here, 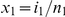, and 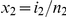. The transition probabilities are given by
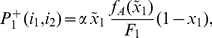
(6)

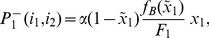
(7)

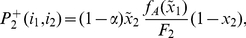
(8)

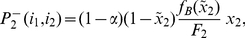
(9)where 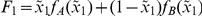, and the equivalent 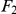, are the average fitness values in each population. The probability that the state 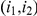 does not change (e.g. when a type 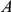 dies and another type 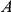 is born) is thus given by 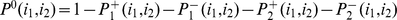. The only trivial boundary conditions are 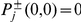, and 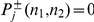 for 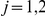. To assess the dynamics of the system, we directly simulate the stochastic process described by Eqs. 6–9.

### Lifetime in an island population close to a continent

The average allelic fitness values in the island population of size 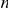 are
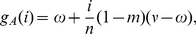
(10)

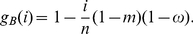
(11)Note here that for the rescaled allele frequency 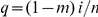, we just have 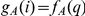, as well as 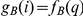, compare Eqs. 3 and 4. The transition probabilities of the one-dimensional Moran process are
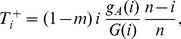
(12)

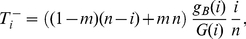
(13)where the normalization (total fitness) is given by 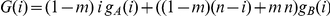.

The parameter transition from high to low migration leads to a change of the local gradient of selection 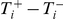, Eqs. 12 and 13. The boundary 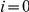 is absorbing, while 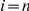 is reflecting,
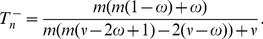
(14)Note that 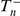 does not depend on the size of the island population. Furthermore, 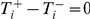 has the trivial solution 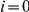, and can have two non-trivial solutions 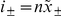, given by
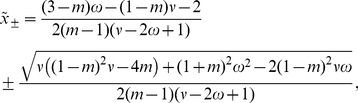
(15)which is real valued if 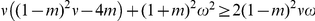. Hence for
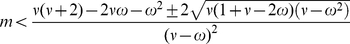
(16)the deterministic one-dimensional dynamics has a stable fixed point at 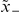, and an unstable one at 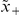.

Let 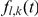 be the probability that the process moves from state 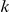 to state 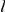 in exactly 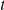 time steps. For this probability function the backward master equation
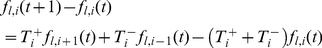
(17)holds, for which we can compute the conditional moments in the following way. The only absorbing state is 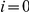, as 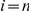 is reflecting, 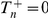, 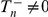 for 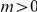. We call 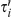 the 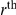 moment of the life time of the process starting from any 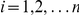. For these moments, the following moment generating recursions hold [31], [33], [38]:
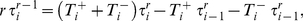
(18)where for the zeroth moment we have 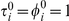, which is the probability that the system fixes at 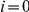 after an arbitrary number of (but at least 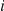) steps. Hence, for the mean life time, 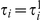, i.e. the first moment of the process, we find
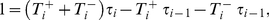
(19)which we can solve recursively. Introducing 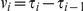, we get
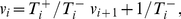
(20)which, respecting the boundary condition and starting from 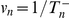, solves to
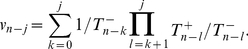
(21)Changing 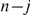 to 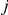 (and the upper limits of sum and product accordingly), we see that 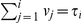, such that the mean life time, starting from any 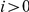, fulfills
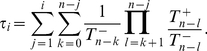
(22)Similarly, all moments follow from Eq. 18, leading to Eq. 5 [33].

## Results/Discussion

### Extinction events in two populations of comparable size

**Figure 3:**
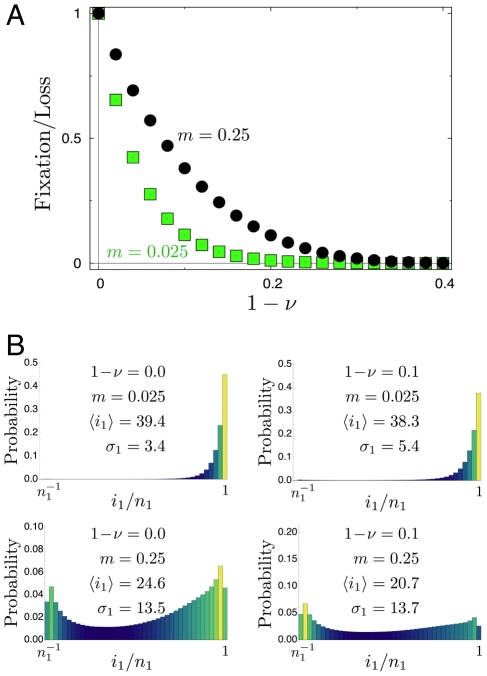
Fixation becomes unlikely with decreasing fitness of mutant homozygotes, variance in allele frequency increases with migration rate. **a**) The ratio of fixation to loss of the mutant allele in a system of two populations of sizes 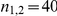 is shown as a function of the difference of homozygote fitness values 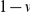, with initial condition 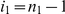, 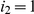. Results are obtained from 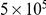 independent realizations with a heterozygote fitness of 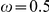. As 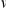 approaches 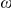, the probability of fixation in both patches goes to zero. **b**) For four different scenarios of homozygote fitness 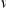 and migration rate 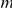 we show the quasi-stationary distribution of the number of mutant alleles in population 1 (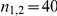, 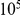 independent realizations with initial conditions 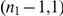). The average number of mutants in population 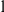 is denoted by 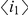, the standard deviation by 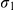. As homozygotes become less fit, the distribution does not change significantly. However, 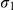 increases with migration rate.

**Figure 4:**
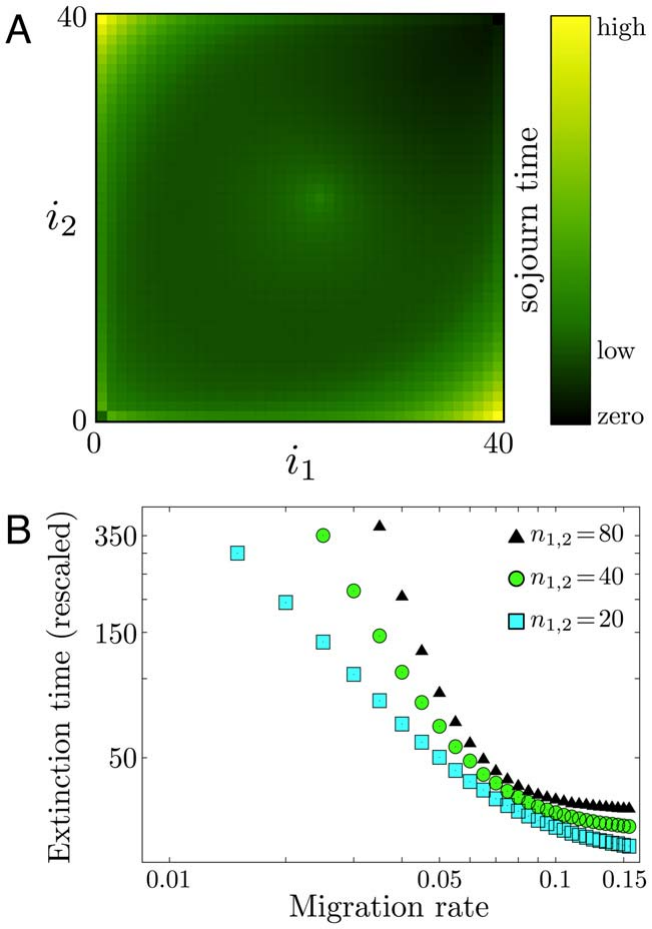
Mutant allele's extinction is delayed for small but non-vanishing migration rates. (**a**) Histogram of the extinction process and the according extinction times as functions of the migration rate in systems of two equally large populations (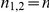, 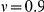, 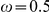, 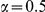). This histogram can be obtained by averaging over sample trajectories such as those shown in Fig. 2. The initial condition is the unstable equilibrium near the center 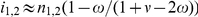, the outcomes are conditioned on extinction (final state 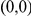). Histogram across the entire state space, 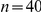, 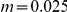 (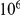 realizations). For each state we give a record of the time spent. Black states are never visited, colored states are visited at least once. The brighter the color, the more often the respective state has been visited, which is characterized by a sojourn time in that state. (**b**) The mean extinction time rescaled by 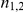, for three different system sizes as a function of 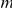, in a double logarithmic plot. Symbols refer to 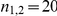 (squares), 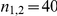 (circles), 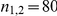 (triangles) (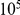 realizations).

First, we address the ratio of fixation to loss in the system of two coupled sub-populations of equal size. An ideal case for a locally controlled genetic pest management strategy emerges when the resistant allele (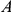) is at high frequency in one local population and at very low frequency in another. Given the situation of almost-all 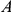 in one population, and almost-no 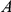 in the other, what is the probability of the allele 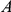 to become extinct in both populations, 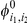, relative to the probability to reach (typically undesired) complete fixation, 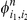
? The answer is given in Fig. 3 (a) showing the ratio 
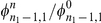
, for 40 alleles in each population, as a function of increasing fitness asymmetry 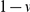, with heterozygote fitness kept constant 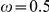. The ratio of fixation to loss of 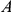 approaches zero with decreasing fitness of homozygote mutants 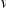. The rate of decay decreases with increasing migration rate, as for low values of 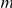 the system spends long times in the interior, compare to the histogram in Fig. 4. In addition, the frequency distribution becomes broader with increasing 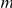, see Fig. 3 (b).

**Figure 5:**
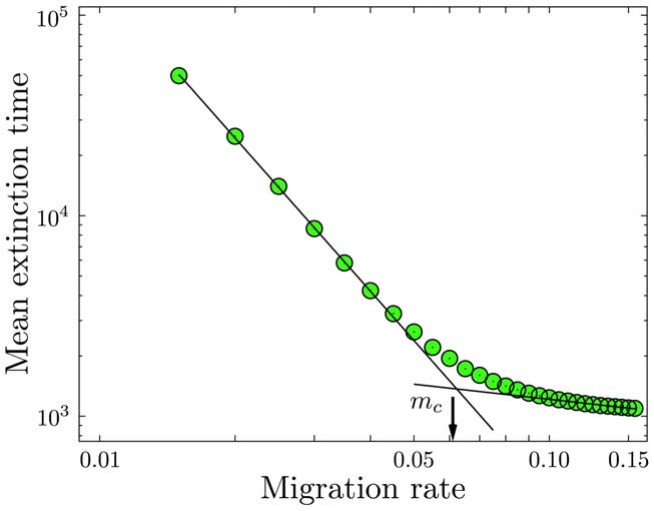
Transition from rapid to slow extinction as migration rate decreases. The mean extinction time as a function of the migration rate (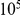 realizations) for 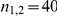, in a double logarithmic plot for mutant homozygote fitness 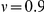 and heterozygote fitness 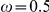. The initial condition is near the deterministic unstable equilibrium 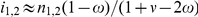. The arrow indicates the value of critical migration rate of the deterministic replicator dynamics, Eq. 2, 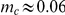. Values for the probability of extinction for the same parameters are 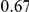 (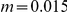) and 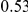 (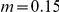).

The replicator dynamics for two populations shows a maximum of nine fixed points and an associated bifurcation pattern depending on the migration rates, see Fig. 1 and compare to [26]. A stable interior equilibrium at migration-selection balance can be disturbed by the demographic fluctuations and will ultimately result in fixation or loss of one of the alleles, despite the stability of the original situation. Hence, one is interested in the average extinction time under various parameter configurations. To grasp an idea of how the system behaves in a single realization, we show three typical stochastic trajectories, Fig. 2. Naively, one would expect the system to spend more time near interior stable equilibria. However, the process spends most of its time in the adjacent edges and corners of the joint allele frequency space, where waiting times are long. The system exits the regions around stable points (e.g., near the 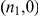 corner) via the edge rather than on internal trajectories, see Fig. 2, because the demographic noise is proportional to 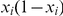 [31]. Hence, in the non-absorbing corners there is little noise and thus we expect long waiting times. Between corners and especially in the interior away from the edges the dynamics are relatively fast. An example histogram of extinction events is given in Fig. 4 (a). For instance, the mean extinction time in a system with 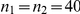 alleles is 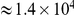 time steps for small 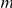. The extinction process spends most of its time near the 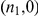 or 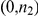 corner. For a very long time the mutant allele is essentially fixed in one population and lost in the other. However, if migration rates become larger, the length of this quasi-stable period decreases (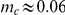 for 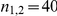), compare to Fig. 5.

The impact of system size in two equally large populations can now be quantified in terms of the average extinction time of type 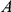. The extinction time diverges with increasing population size. Fig. 4 indicates that for lower migration rates, this effect is stronger. Low migration, 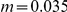, gives an average extinction time of approximately 30000 time steps, which amounts to approximately 375 generations in a populations of 80 alleles. For high migration, 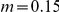, we obtain an average of approximately 2500 time steps (approximately 31 generations). This number of generations is consistent with the expectation that the two populations become panmictic for high migration: In a panmictic population of 160 alleles, the standard literature on Moran models [33], [39] yields an average extinction time of approximately 2500 time steps (approximately 16 generations).

Analyzing the extinction times as a function of migration rate reveals the transition from one power law to another in the region around the critical migration rate predicted by the replicator system; We can identify two regimes. In the first regime, 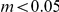, the extinction time scales as 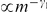, with 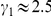. In the second regime, 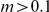, the extinction time scales as 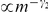, with 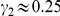. The two power law regimes for 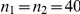 are given in Fig. 5 for a realistic choice of genotypic fitness values 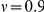 and 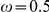. For this parameter configuration the replicator equation 2 yields a critical value of 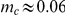 [26]. Fig. 5 analyzes this transition for 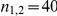. The initial condition is chosen such that 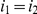 are at or close to the selection-migration equilibria, 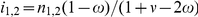, which is near the most efficient release strategy in terms of minimal release numbers (discussed below).

### Temporary maintenance of polymorphism in an island population

**Figure 6:**
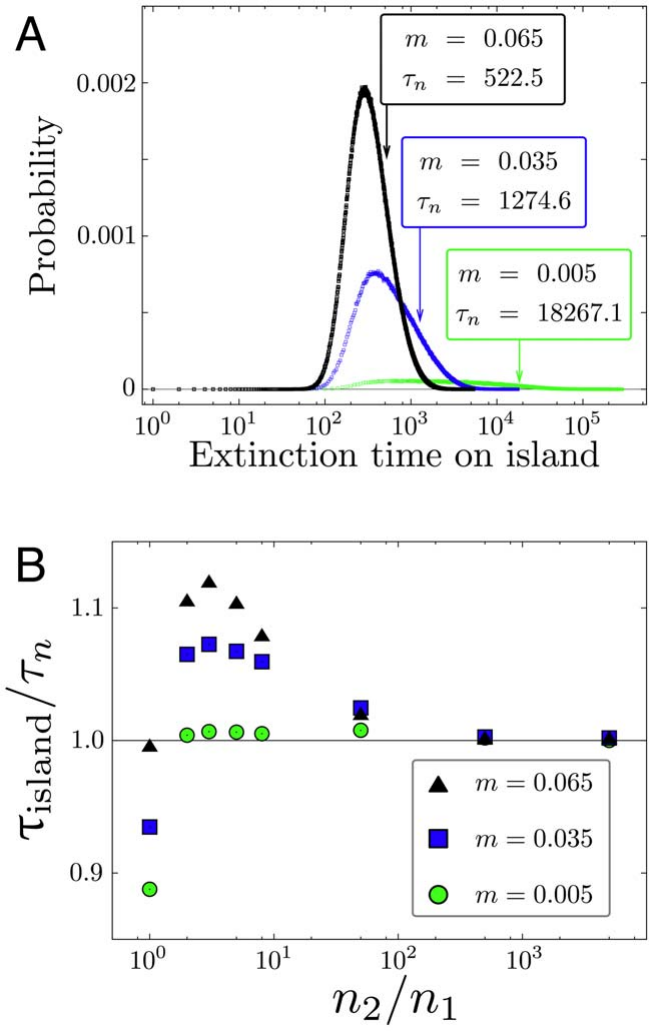
Maintenance of polymorphism in a small island population. **a**) Histograms of the extinction time on an island population, Eqs. 12,13, for different migration rates in a log-linear plot. Population size is 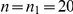, fitness of mutant homozygotes is 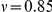, fitness of heterozygotes is 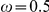. The histograms stem from 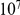 independent realizations with initial condition 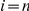. Each arrow indicates the mean extinction time 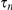, Eq. 22 (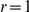). The values from simulation and the exact formula are in excellent agreement. With decreasing migration rate, the distribution of extinction times broadens significantly. **b**) For the same set of parameters we show how the (conditional) average extinction time of the mutant allele in a small population converges to the analytical result of the continent-island approximation with 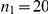 and variable 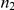 (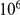 independent realizations).

The transition of one population approaching infinite size, while the other remains relatively small, leads to stochastic evolutionary dynamics in one dimension. A benefit in using a Moran model is that in one dimensional systems we can obtain exact analytical results for the hierarchy of moments of extinction/fixation times [31], [33], [38], [39]: We can solve the recursions for the moments, Eq. 18. In Fig. 6 we present the convergence of the limit 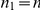, 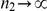 (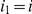, 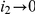) and show histograms from simulations of the one-dimensional island model, Eqs. 12 and 13. The distribution of extinction times changes substantially with 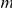. In our example, for very low migration rates the mutant allele is expected to be maintained in the system for more than 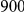 generations, when starting from 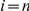. With a fixed population ratio, we average over the change of the Moran process in the island population to obtain a measure 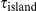, discarding changes in the continent population. As the ratio 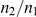 increases, this average converges to the average extinction time 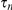: The simulations start from 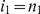, 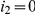, and with increasing 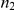, fluctuations in the continent population decrease, 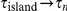. Only for a continent population which is roughly a hundred times larger than the island population, we enter the regime of a quasi one-dimensional system with a static continent of wildtypes. The limit case is not approached monotonically, but depends on the migration rate 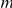 in a non-trivial way.

### Release strategies and probability of long term transformation

**Figure 7:**
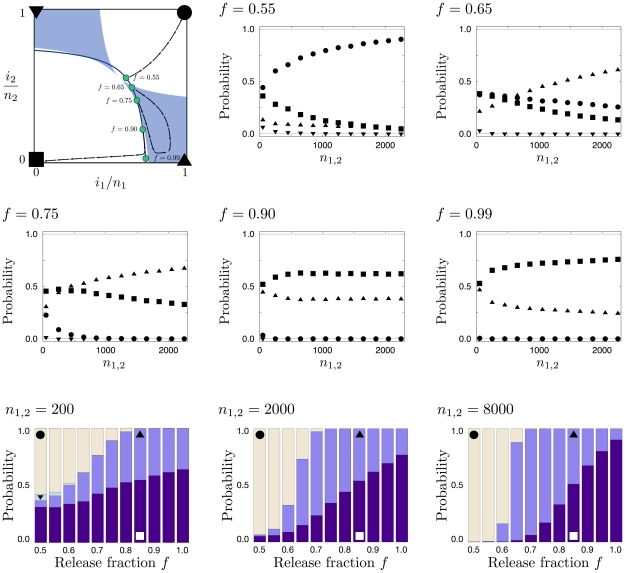
Searching for an optimal release strategy. The upper left panel illustrates the deterministic basins of attraction for 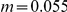, 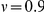, and 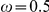. The blue line illustrates possible starting points for a release of size 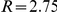 for all possible values of the release fraction, 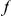, into population 1. Blue disks correspond to points of illustration in the five following panels. The arrow streams represent example trajectories of deterministic dynamics. The following five panels are labeled according to the release fraction 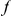. Symbols correspond to the probability of reaching the correspondingly labeled corners (in the upper left panel) and indicate how they change with 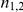. Although complete fixation or loss are the only possible long term events, there is a probability that the neighborhood of, e.g., 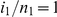, 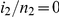 is reached first, which we refer to here by triangles. In particular, note that the probability ranks interchange at certain population size for 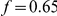 and 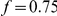. The three bottom panels, labeled with the respective system sizes, show the corner probabilities as a function of 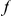. A release strategy with 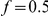 maximizes the likelihood of transforming both populations. In contrast to that, 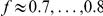, maximizes the likelihood of transforming only a target local population. Higher values of 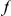 then proceed to an increasing likelihood of rapid loss in both populations. All results are obtained from 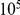 independent realizations.

Assuming migration is low enough such that it can be locally counteracted by selection, how likely is a mutant allele to fix or be removed depending on the initial transformation? Here, we give a characterization of the two population system in terms of complete loss or fixation in both, or temporary reciprocal fixation and loss. In principle, a release strategy is based on two parameters. First, the amount of new mutant alleles added to the system, 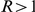, relative to the size of each population. Second, a fraction 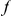 is released into one, and the remainder 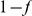 into the other population. Maintaining the populations at a constant size implies that
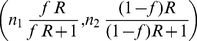
(23)is the set of initial release points, where 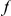 ranges from 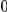 to 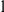. For a given 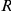, this defines a curve in the joint allele frequency space, which is depicted in Fig. 7 for different values of 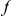. Also in Fig. 7 the probabilities of first visiting a corner of the system for a fixed release size 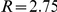 and various fractions 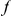 are summarized as a function of the population sizes 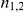. In accordance with Fig. 3, the system loses the mutant allele entirely with a high probability for a wide range of chosen release strategies.

Fig. 7 illustrates some less intuitive properties that are informative in terms of release strategies. For a given number of genetically modified individuals 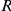, it might seem that releasing all of the individuals into a single target population would maximize the chance of successfully transforming the population. However, in this case, simultaneously releasing some individuals into the neighboring population is more likely to result in a successful local transformation. This proportion is dependent on the population size, 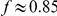 for 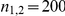 to 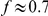 for 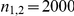 or higher. To understand this dependency, note that the basin of attraction of the local transformation is a smaller proportion of the local space near the central unstable equilibrium. Since the demographic noise in finite populations is proportional to 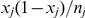, the basin of interest comprises a smaller proportion of states where selection can be counterbalanced by local migration. In the illustrative example in Fig. 7 it can also be seen that a simultaneous equal release into both populations (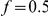) maximizes the chances of transforming both. Attempting to transform one population at a time in a stepwise strategy does not lead to complete fixation immediately. However, once a single population is successfully transformed, it is much easier to transform the neighboring population, if desired. This only requires an additional release of less than a single population size, 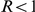.

### Summary and conclusions

We have proposed a simple model to analyze the influence of small system size and system size asymmetry on the evolutionary dynamics of an underdominant system in a structured population. The population structure itself is chosen to be as simple as possible: We consider two sub-populations that exchange migrants at a given rate. This allows a direct comparison with findings in infinitely large populations [24], [26]. Our simplifying assumptions then permit a statistical characterization of the migration-selection equilibrium in finite populations by means of simulations. Other stochastic descriptions of the evolutionary dynamics, e.g., the Wright-Fisher process, have very similar properties when it comes to extinction probabilities and times [31], [40], [41]. However, we stick to a Moran model which has the benefit that limit cases have exact solutions for all fitness values and population sizes that do not rely on further approximations.

We review previous findings in infinitely large populations in the introduction and use them as a basis to examine the influence of demographic fluctuations in small populations. We argue that the transient dynamics are important, as the influence of noise may alter the outcome of the evolutionary dynamics in this regime.

Our main results can be summarized as follows. First, for fitness asymmetry, extinction rather than total fixation of the potentially underdominant allele is the most likely outcome, even if this allele is initially at high frequency in one of the populations. In a migration-selection equilibrium the (quasi-stationary) variance in allele frequencies is low. High migration disrupts this dynamic equilibrium such that extinction is facilitated and the variance in frequency increases.

Second, we find that migration rate has a strong impact on the extinction process. We identify a threshold below which the mutant allele can be maintained for a long time, which corresponds to a bifurcation point in the deterministic system. For example, if mutant homozygotes suffer from a 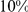 fitness loss and heterozygotes from a 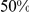 fitness loss (compared to wildtypes), extinction is significantly delayed for migration values below 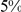. With increasing population sizes, the extinction times tend to diverge rapidly with decreasing migration in this regime. Even in small populations, disruptive effects from demographic fluctuations can be counterbalanced by small, but finite numbers of migrants.

Third, we evaluate the consequences for release strategies. For conservative estimates of a release of potentially underdominant mutants into wildtype populations, we can give a statistical evaluation that can be tested *in vivo*, as well as *in situ*. If migration between patches is low enough, a release division of 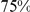 mutants into a target population, and thus 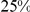 into a neighboring population, can be optimal and lead to a local polymorphism that is expected to be maintained for a long time.

Fourth, the limit case of one population becoming very large reveals that the potentially underdominant allele can be kept in the small population for long times. A small population with incoming migrants from a large wildtype reservoir is well described by a one-dimensional process if the reservoir is about 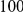 times larger. This also refers to the desired situation in which one is interested in the local establishment of disease resistance (caused by an effector gene), driven by underdominance.

Results from infinite population assumptions may, in some cases, be misleading when observing finite allele frequencies. Under demographic fluctuations the stochastic evolutionary dynamics slow down near corners and along edges: In the vicinity of equilibrium points the flow induced by selection can become squeezed between boundary and equilibrium. High flow density means low flow velocity, which also affects the transition rates. Due to this nature we may observe large waiting times near the corners and along edges which happen to be near internal equilibria. However, under neutral evolution, the system also slows down near corners and edges.

If selection is strong, underdominance and sufficiently low migration can maintain a polymorphic state for many generations even in small populations. This bodes well for using underdominance to control initial testing of genetically modified insects in isolated settings so that the natural species remains untransformed in its broader range. The system can be stable for so long that additional factors are likely to be more important in ultimately disrupting the system. Such additional factors can be the occurrence of new mutations and/or behavioral changes [42], [43], [44].

The stability properties of underdominance in small finite populations may have particular value both in initial testing of genetically modified vectors and in species conservation applications. For example, *Culex quinquefasciatus* mosquitoes have spread from a native range in the southeastern United States to several islands in the Pacific due to human activities. Most of these islands are of substantial conservation value, e.g., the Galápagos [45] and Hawaiian archipelagoes [30]. The mosquitoes are vectors of avian malaria, *Plasmodium relictum*, which is a major factor in past extinctions and current endangerment of many Hawaiian forest birds [30]. Island populations of *C. quinquefasciatus* can be genetically modified to be refractory to avian malaria to break the cycle of infection, e.g., [46], [47]. Linking this refractoriness to underdominance could prevent the genetic modifications from spreading back into the native range of *C. quinquefasciatus*. This would allow the native range mosquitoes to be protected in a wildtype state. Furthermore, it may also be possible to leave a fraction of the island populations stably untransformed to allow the evolution of natural resistance in the threatened bird species (see, e.g., [48]).

Genetically modified chromosomes are typically less fit than wildtypes as homozygotes, see [49] and references therein. This homozygote fitness asymmetry provides a degree of failsafe into the system. If stability is lost, the system is much more likely to result in a return to a natural wildtype state, rather than reaching fixation of an artificial genetic modification across populations.
